# Honey Pollen: Using Melissopalynology to Understand Foraging Preferences of Bees in Tropical South India

**DOI:** 10.1371/journal.pone.0101618

**Published:** 2014-07-08

**Authors:** Raja Ponnuchamy, Vincent Bonhomme, Srinivasan Prasad, Lipi Das, Prakash Patel, Cédric Gaucherel, Arunachalam Pragasam, Krishnamurthy Anupama

**Affiliations:** 1 Department of Ecology, French Institute of Pondicherry, UMIFRE 21 CNRS-MAEE/USR 3330, Pondicherry, India; 2 Department of Botany, Kanchi Mamunivar Centre for Post-Graduate Studies, Pondicherry, India; 3 Sri Aurobindo International Centre of Education, Pondicherry, India; Universidade de São Paulo, Faculdade de Filosofia Ciências e Letras de Ribeirão Preto, Brazil

## Abstract

The aim of the study was to use melissopalynology to delineate the foraging preferences of bees in tropical environs. This was done by comparing pollen spectra obtained from the same hives every three months for three years at four sampling locations (in two sites) within a confined landscape mosaic. If melissopalynology is highly replicable, the spatial variation of the pollen spectrum from the honey samples would be much more than the temporal (inter-annual) variations. In other words, given the three factors, Month, Year and Location, honey pollen from different Locations, in a given Year and Month, would be much less similar than samples from different Years, in a given Location and Month. We then determined how the factors, Month, Year and Location, influenced the pollen influx of honey. The pollen analyses of the 42 honey samples collected during the three years yielded 80 pollen taxa/types: 72 dicotyledonous and 8 monocotyledonous, encompassing 41 botanical families spread into seven life forms namely, trees, shrubs, epiphytes, herbs, climbers, grasses, and sedges. Our results showed that pollen spectra were equally comparable between Locations and between Months and Years; the importance of this result is that it helped to demonstrate the complexity of ecological/environmental phenomena involved in the process of foraging by bees in a heterogeneous and complex landscape.

## Introduction

Bees, the primary pollinators of the world, play a crucial role for wild and cultivated plants, especially in the tropics where insect pollination is vital [1]–[2]. Melissopalynology is the study of the pollen they collect, intentionally and accidentally, which gets into honey. Melissopalynology has been extensively used [3]–[10] to determine the purity, geographical and floral origins of honey. It is also used to assess correlations with *in situ* climatic parameters such as rainfall and temperature [11]–[12] important in the context of external factors influencing pollinators and pollination networks [13]–[19]. Statistical analyses, mainly ordination, have been carried out on melissopalynological data in quantitative studies to obtain more robust characterization of the honeys in terms of their geographic and botanic origins [20]–[22].

The many variables that come into play in the production of honey, including the ability of honeybees to remove certain types and certain amounts of pollen from the nectar they collect prior to returning to the hive, have been summarized [23]. Though acknowledged that the complexity introduced by these different variables implies a substantial variation from year to year or season to season in terms of the pollen contents of honey produced in the same hive [23], to our knowledge, this replicability, *i.e.*, stability in time and space, has not been tested statistically. This study attempts to fill the gap using the primary data generated in the course of a pluri-annual melissopalynological survey near Puducherry, South India.

The aim of the study was to use melissopalynology to delineate the foraging preferences of bees in tropical environs. There are several methods, including direct observations and video monitoring (11,14,18–19) to understand the foraging preferences of bees. Analyzing pollen in honey provides a complementary, robust, rapid and quantitative method to do this. This was done by comparing pollen spectra obtained from the same hives every three months for three years at four sampling locations (in two sites) within a confined landscape mosaic. Our hypothesis is that if melissopalynology is highly replicable, the spatial variation of the pollen spectrum from the honey samples would be statistically much more than the temporal (inter-annual) variations. In other words, given the three factors, Month, Year and Location, honey pollen from different Locations, in a given Year and Month, would be much less similar than samples from different Years, in a given Location and Month. We then determined how the factors, Month, Year and Location, influenced the pollen influx into honey.

Here, we test the replicability of melissopalynology for the first time in the tropics and delineate the foraging preferences of bees and provide methodological recommendations for further melissopalynological experimental designs.

## Materials and Methods

### Ethics statement

The study was conducted in privately owned areas where prior permission was obtained: site 1 to which two of the authors (LD and PP) are affiliated, and site 2 managed by Bernard Declercq and Deepika Kundaji, Auroville. The study did not include any human subjects or vertebrate animals, and honey was collected with minimal disturbance to the honeybees.

### Study site

**Figure 1:**
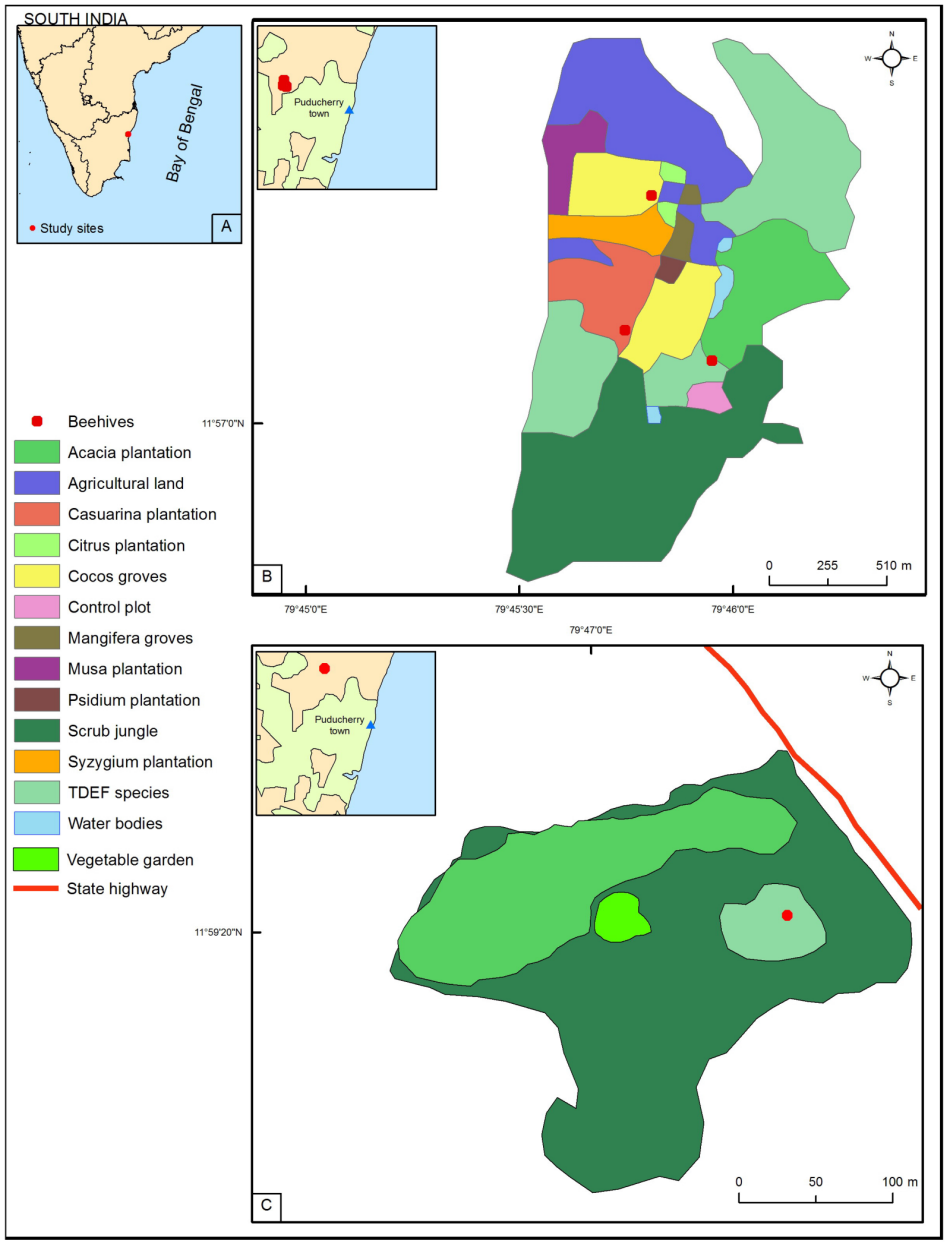
Location detail and mosaic vegetation of study areas. A. South India with state boundaries, B. First three locations in site 1 with surrounding vegetation C. Fourth location in site 2 with surrounding vegetation.

The study was conducted in four locations in two sites that are 6 km apart. The first three locations were in site 1, spread over 160 ha at 40–50 m a.s.l. (11°57′8.3″ N, 79°45′57.2″ E). Location 4 was in site 2 (11°59′20.4″ N, 79°47′4.2″ E and 50 m a.s.l.), spread over 10 ha (Figure 1).

In the Puducherry region, rainfall is distributed over a part of the summer and a part of the (tropical) winter, *i.e.*, from July to January. Much of the rain occurs during the northeast monsoon between October and December (records from Puducherry weather station). Mean temperature in December was 25°C for the years from 1911 to 1961 [24]. In the last three decades (1980–2009), the pattern remained similar.

### Bee species, beehive locations, and surrounding vegetation

As part of an ongoing eco-restoration project, particular effort was made to rear the Asiatic honeybee *Apis cerana* Fabricius, one of the native honeybees of India [25]. The bees were reared in wooden beehives placed in four locations. The locations were chosen to reflect the complex mosaic of vegetation (Figure 1; [26]). One beehive per location was considered in this study.

The four locations are Garden and Tropical Dry Evergreen Forest (GT), Coconut Grove (CG), Agricultural Field (AF), and Scrub Jungle (SJ). GT contains ornamental plants, several introduced drought-tolerant *Acacia* spp., *Casuarina junghuhniana* Miq., small patches of restored Tropical Dry Evergreen Forest (TDEF) with characteristic species like *Memecylon umbellatum* Burm. f. and *Allophylus serratus* (Roxb.) Kurz and scrub thicket elements like *Benkara malabarica* (Lam.) Tirveng. and *Ziziphus oenoplia* L. (Mill.). CG comprises a mixed plantation of *Cocos nucifera* L., *Casuarina junghuhniana* Miq. and *Manilkara zapota* (L.) P. Royen, where cereals are intercropped during the monsoon. AF comprises cereals, vegetables, pulses, and tubers, cultivated by usual crop rotation practices, and surrounding plantations of *Citrus* spp., *Cocos nucifera* L., *Mangifera indica* L., *Musa paradisiaca* L., and *Psidium guajava* L. Local herbaceous plants like *Trianthema portulacastrum* L. and *Waltheria indica* L. are common in the CG and AF. The dominant species in SJ are characteristic of scrub jungles: *Benkara malabarica* (Lam.) Tirveng, *Catunaregum spinosa* (Thunb.) Tirveng., *Dodonaea viscosa* (L.) Jacq., *Glycosmis mauritiana* (Lam.) Tanaka, *Flacourtia indica* (Burm. f.) Merr., *Securinega leucopyrus* (Willd.) Muell.-Arg., *Toddalia asiatica (L.)* Lam. var. *gracilis* Gamble, and *Ziziphus oenoplia* L.

### Sampling of honey and identification of pollen content

Samples were collected seasonally in the fourth week of February, May, August, and November during 2007, 2008, and 2009 in the four locations, later referred to as the independent variables Month, Year, and Location. A contiguous 10 cm×10 cm area containing pollen cells as well as honey cells from a brood comb was chosen for (pseudo-random) collection. Each sample was collected by squeezing out honey along with pollen without harm to the bees. The honey and pollen samples were collected in sterile containers by two of the authors (LD, RP) exclusively for the melissopalynological studies. We retrieved 42 honey samples; six samples could not be retrieved due to hive abandonment.

**Figure 2:**
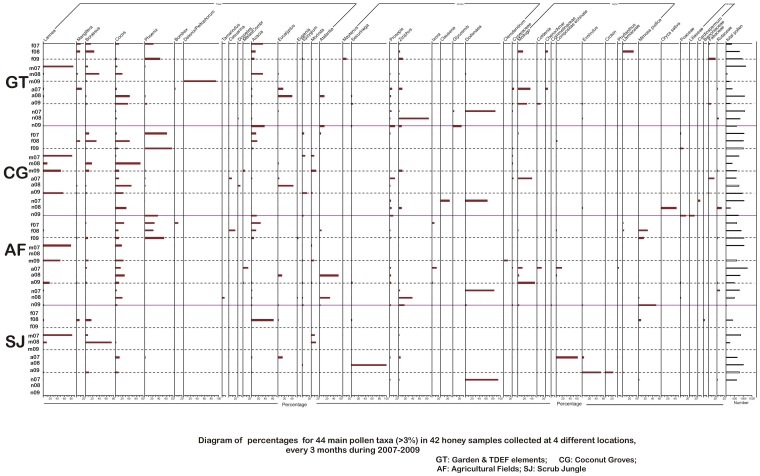
Pollen diagram showing the percentages of the main taxa and the total pollen sum (f =  February; m =  May; a =  August; n =  November; 07 = 2007; 08 = 2008; 09 = 2009).

**Figure 3:**
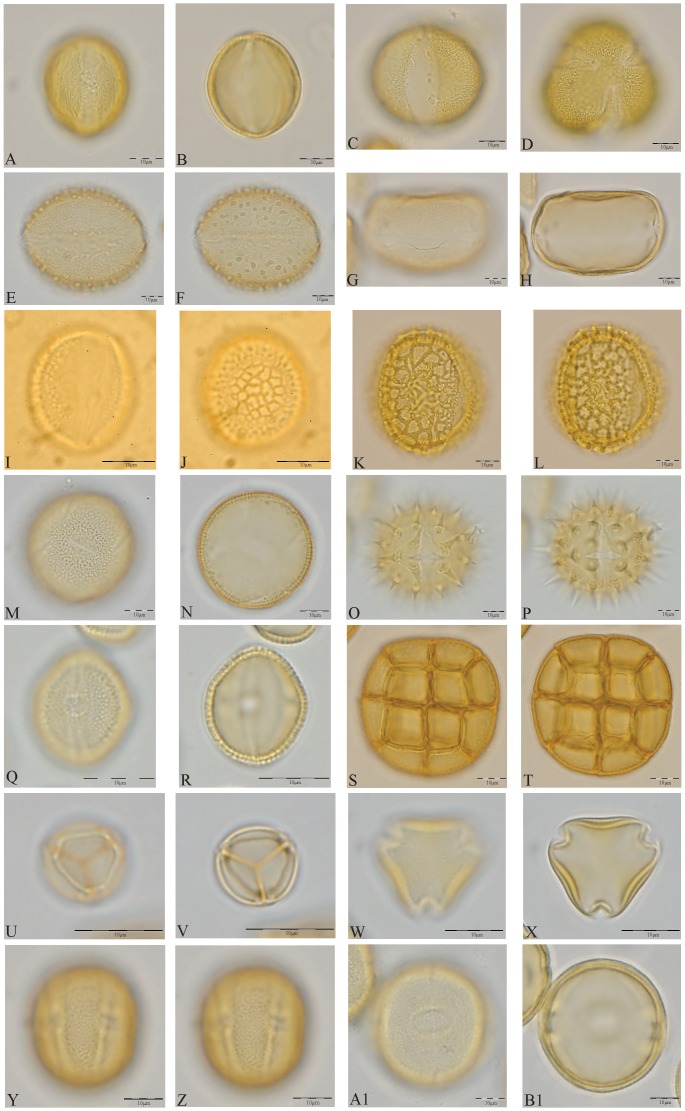
Photomicrographs showing the 14 predominant pollen types *i.e.*, uni-floral origin (a single pollen type represented >45% of total observed pollen types in a sample) in the 42 honey samples arranged ascending order of family: A–B *Lannea*; C–D *Mollugo*; E–F *Borassus*; G–H *Cocos*; I–J *Phoenix*; K–L *Delonix/Peltophorum*; M–N *Evolvulus*; O–P Compositae-echinate; Q–R *Securinega*; S–T *Acacia*; U–V *Mimosa pudica*; W–X Rhamnaceae; Y–Z *Atalantia*; A1–B1 *Dodonaea*.

Honey was dissolved in warm, distilled water to remove sugars and water soluble components and centrifuged. The supernatant was discarded with care to check for loss of pollen during this procedure. The residue was then processed for pollen studies using standard acetolysis method [27], mounted in glycerine medium and observed under ×500 magnification using a Light optical microscope (Wild M20). Pollen morphological features, as revealed by the qualitative and quantitative features of the outer wall [28]–[30], formed the basis for the identification and enumeration of pollen types [31]–[32]. They were identified and enumerated using the reference pollen slides of the French Institute of Pondicherry as well as pollen floras [33]–[36]. High pollen counts, averaging 6933±3097 were obtained, keeping in mind both tropical diversity and the under- and over- representation of pollen in honey [23]. Pollen frequency classes were assigned using standard procedures [37]. The primary data generated in the course of this study is represented as a pollen diagram (Figure 2) drawn using TILIA [38] and a plate of pollen photomicrographs (Figure 3).

### Statistical analysis

All statistical analyses were done using the statistical environment R 2.15.1 [39]. Similarities between pollen spectra were computed using two indices: binary Bray-Curtis' index (bBC) and Bray-Curtis' index (BC). These two indices compare pollen presence/absence and pollen frequencies between samples, respectively, and are calculated as follows [39]:
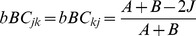
and
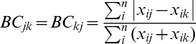



For the bBC index, *A* and *B* are the numbers of species in samples *j* and *k*, and *J* is the number of species found in the two samples. The BC index uses the absolute distance between *x_ij_* and *x_ik_*, which are pollen frequencies for the *i* ^th^ over *n* species in samples *j* and *k*.

Multivariate analyses of variance (MANOVA) were performed to test the effects of the independent variables Month, Year and Location on the pollen spectra, the latter being the dependent variable since we aimed at testing if the pollen spectra vary between Months between Years, and between the Locations and if any interaction between these factors exists in our dataset.

We nested Month and Year factors within the Location factor since their repetitions are meaningful only within the same location. For model selection, as no information-based criterion (e.g. Akaike information criterion) is available for MANOVA performed on distances matrices, we used the vegan package [39] and 10^5^ permutations to estimate the distribution of the statistics. Then all interactions starting from the full model have been tested; backward procedures were adopted for model selection, with the successive removal of the interactions of the highest order with an arbitrary threshold *α* = 0.05. Forward selection led to the same final models. Distance matrices and their graphical representation as heatmaps were built using the pair-wise distances for the two indices and between samples.

Principal Component Analyses (PCA) explored how the structure of our experimental design (Month, Year, Location) interplay in the total variance of our dataset as summarized by the principal components. In other words, if the replicability is high among sites, we expect samples of each site to cluster together, no matter which Year or Month they were collected. Then, Linear Discriminant Analyses (LDA) with and without cross-validation (leave-one-out) determined the classification error, i.e., the value of previous knowledge. In other words, if for every sample, we use the grouping structure of all the samples collected but one, and then introduced the left out sample, do we classify it correctly? PCA was performed and illustrated using the ade4 package [40] to explore how pollen taxa and different Months, Years, and Locations are distributed. Confidence ellipses (±1.5SD on every PC), corresponding to the categories of every factor and drawn a posteriori on the factorial map, help graphically to understand the patterns observed. Scatter correlation circles were then used to explore and attribute the influences of each pollen taxon. Percentages of the variance explained by the successive PC axes were calculated using the Eigen values of the variance/covariance matrix. LDA was performed using the package MASS [41] to test whether the discriminant linear combinations of pollen taxa were able to discriminate between groups. The standardized discriminant coefficients were used to compare the relative importance of the pollen taxa in order to discriminate within/between groups of samples. Three LD axes were obtained for Month and Location (4 levels each) and two LD axes were obtained for the factor Year (3 levels each).

The LD axes that brought the maximum between-groups differences were considered for each of the factors. LDA was first performed on the complete dataset of 80 taxa. We then tested if LDA can also be used in our case as a tool to classify additional pollen spectra based on this discriminant function, using the leave-one-out cross-validation implemented in the MASS package [41]. The idea behind this validation is simple: every pollen spectrum is successively removed from the dataset and an LDA is performed and then used to classify the pollen spectrum removed. For the final analyses, we retained only the 51 taxa that occurred at least 3 times in our dataset of 42 samples.

## Results

### Honey pollen content

The pollen analyses of the 42 honey samples collected during the three years yielded 80 pollen taxa/types: 72 dicotyledonous and 8 monocotyledonous, encompassing 41 botanical families spread into seven life forms namely, trees, shrubs, epiphytes, herbs, climbers, grasses, epiphytes and sedges (Table S1). On average, 6933±3097 pollen grains (average ±1 SD are given throughout the text) were counted (min = 283, max = 12356, median = 7591), and 18±7 pollen types (min = 5, max = 30, median = 18.5) were identified per sample.

Three families accounted for more than half the total number of pollen grains: Arecaceae (29%), Anacardiaceae (14%), and Mimosaceae (11%). Arecaceae was present in all, but one, samples. The three most abundant life forms, expressed as percentages of the total pollen count, were trees (39%), shrubs (24%), and herbs (20%). Forty-four out of 80 taxa were recorded at ≥3% of the total (Figure 2). The graph at the extreme right in this figure shows the total number of pollen counted in each sample. Twenty-six honey samples were found to be uni-floral (one pollen taxon occurring at ≥45% of the total pollen count per sample), represented by 14 predominant pollen taxa (Figure 3). As expected, pollen spectra and predominant pollen taxa varied in space and time, a few examples being *Lannea*, *Dodonaea, Phoenix, Borassus*, and *Cocos* (Figure 2).

### Statistical analyses

We performed MANOVAs on the distance matrices calculated using the two similarity indices (Table 1 & Table 2). All the tested factors were highly significant and hence the pollen spectra were not highly replicable.

**Table 1:** MANOVA table for binary Bray-Curtis' distance.

MANOVA on binary Bray-Curtis' distances						
Response variable: Bray-Curtis' distances	df	SSQ	MSQ	F	R^2^	Pr(>F)
Year	2	1.3910	0.6955	4.3182	0.0906	**<0.0001**
Location	3	1.1849	0.3950	2.4522	0.0772	**<0.0001**
Month	3	4.5184	1.5061	9.3511	0.2944	**<0.0001**
Year × Location	6	1.5822	0.2637	1.6373	0.1031	**0.0021**
Year × Month	6	3.2889	0.5482	3.4033	0.2143	**<0.0001**
Residuals	21	3.3823	0.1610		0.2204	
Total	41	15.3477			1	

**Table 2:** MANOVA table for Bray-Curtis' distances. df, SSQ, MSQ stand for degrees of freedom, sum of squares, mean squares.

MANOVA on Bray-Curtis' distances						
Response variable: Binary Bray-Curtis' distances	df	SSQ	MSQ	F	R^2^	Pr(>F)
Year	2	0.7887	0.39435	3.4524	0.1091	**0.0002**
Location	3	0.7635	0.25450	2.2281	0.1056	**<0.0001**
Month	3	1.6813	0.56044	4.9065	0.2326	**<0.0001**
Year × Month	6	0.9098	0.15163	1.3275	0.1259	0.0637
Residuals	27	3.0840	0.11422		0.4267	
Total	41	7.2273			1	

Only the final models are shown.

**Figure 4:**
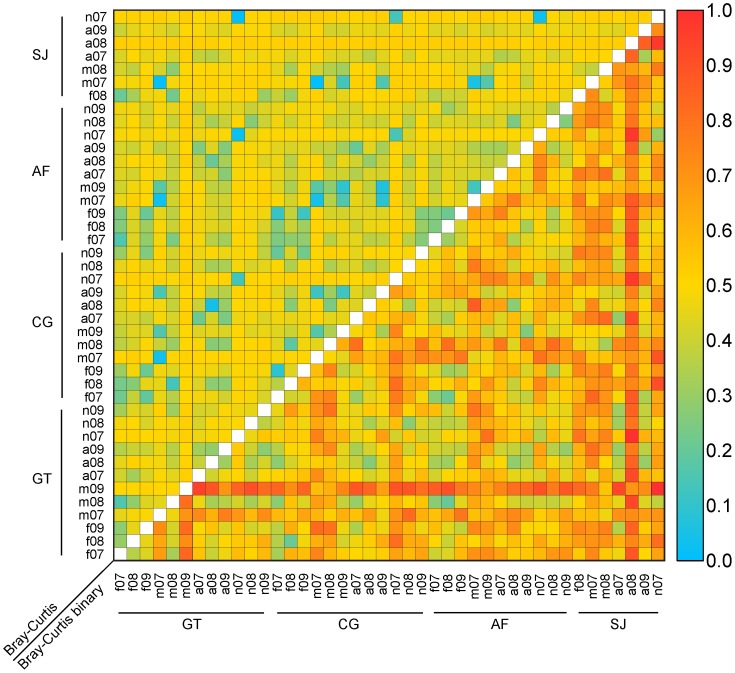
Pair-wise comparison of qualitative and quantitative melissopalynology for pollen similarity studies.

The average Bray-Curtis (BC) index was 0.3 and the average binary Bray-Curtis (bBC) index was 0.4, graphically shown as heatmaps (Figure 4). Taken individually, each factor was highly significant (*P*<10^−4^) and retained in the final model. Only the two interactions Year × Location (*P*<10^−3^) and Year × Month (*P*<10^−4^) were significant in the bBC model; only Year × Month (*P*<10^−2^) was retained in the final BC model (Tables 1a & 1b). The heatmaps showed accordingly no well-defined patterns: when samples were arranged location-wise, there were no higher similarity values along the matrix diagonal (Figure 4), as expected. The only “structure” was that the least similar sample pairs (blue and shades of blue) correspond to samples in different locations in a given year and month (Figure 4, BC model). Similar heatmaps were obtained when arranged month-wise and year-wise.

**Figure 5:**
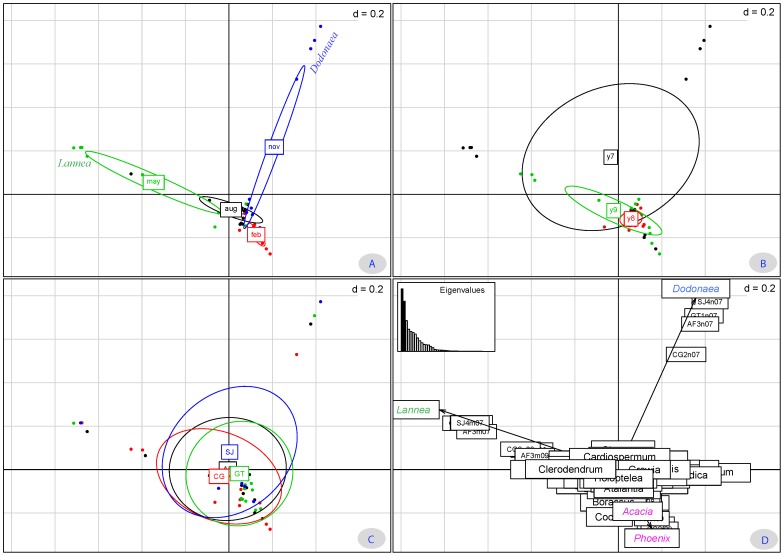
Multivariate analyses (PCA) showing the structure of pollen spectra in reduced dimensionality of absolute pollen frequency. A =  Month-wise, B =  Year-wise, C =  Location-wise, and D =  Percentage of Eigen value and overall variance distribution.

Only a few highly abundant taxa (*Dodonaea*, *Lannea*, *Phoenix*, and *Acacia*) appeared distinctive in the plots on the first two PC axes. The first two PC axes captured 38% (PC1 = 21%; PC2 = 17%) of the total variance (Figure 5D). The graphical relative distribution of the pollen taxa showed two trends on the plot using PC1 and PC2 as principal axes (Figure 5A–D). Moreover, *Dodonaea* and *Lannea* were pronounced on the two directions along two PC axes. First, a clear Month effect was identified (Figure 4A). Then, in the factors Year (Figure 5B) and Location (Figure 5C), such grouping was not as pronounced. In addition, most multi-floral honey samples overlapped.

**Figure 6:**
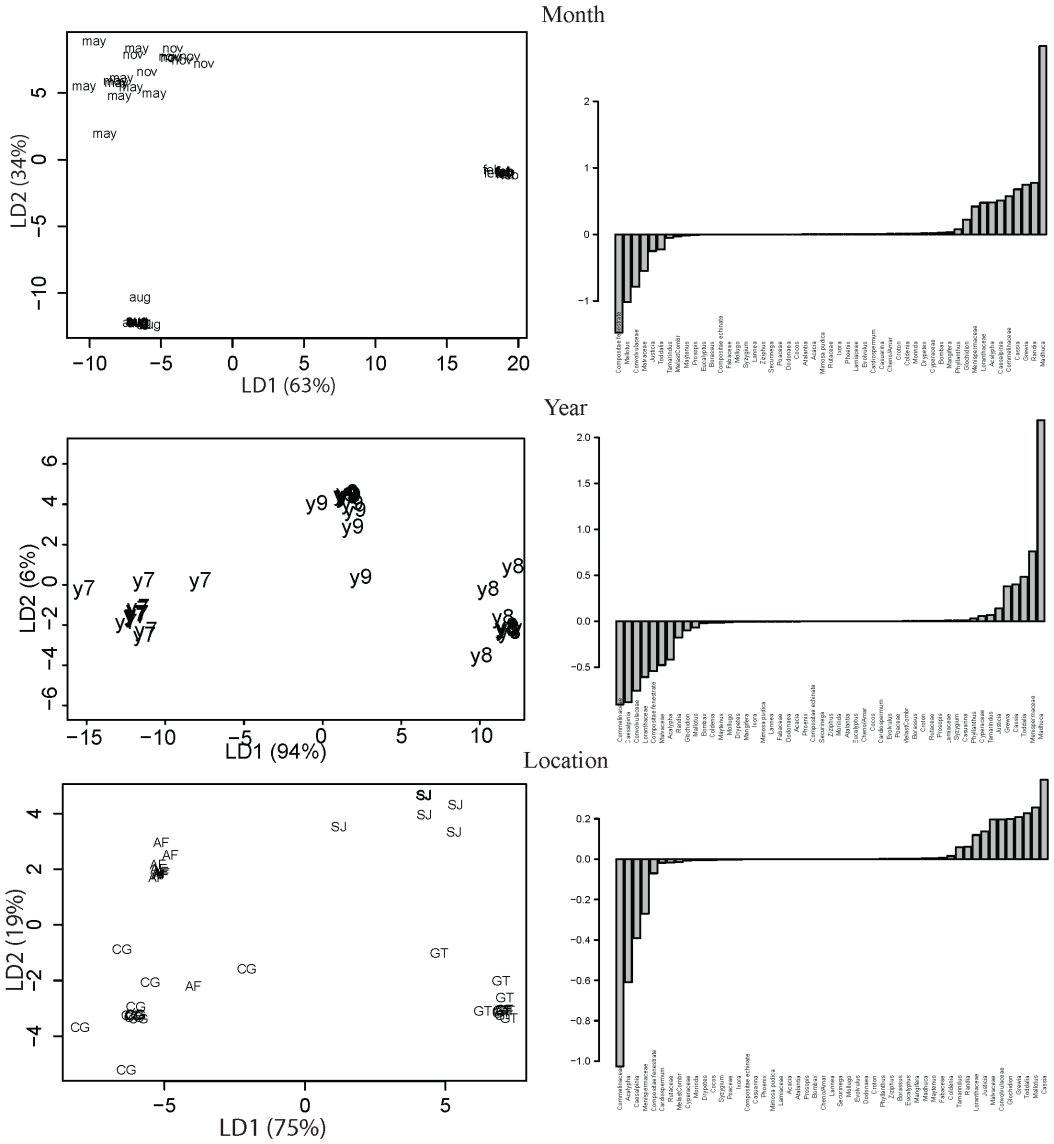
Multivariate analyses (LDA) showing the group membership of honey with reference to spatio-temporal factor and discriminant coefficient value of individual pollen type. For Month and Location, the third LD axis did not bring major between-groups differences; therefore only the first two axes have been retained.

In the final LDA, retaining only 51 taxa, samples were well classified with reference to all three factors (Figure 6). The discriminant coefficient values were high for 15 taxa with reference to one or more factors (Figure 6). *Madhuca* was identified as the most discriminant taxa for Month and Year and, overall, the taxa with the highest discriminant value. Other taxa with high discriminant values for all factors were *Cassia*, *Grewia*, Commelinaceae, and Malvaceae. Most of the taxa with high discriminant power were “low abundance” taxa. The discriminant coefficient values were low (<0.01) for the remaining 35 taxa (Figure 6). Several of the set of taxa with coefficient values closer to zero corresponded to “high abundance” taxa (*e.g.*, *Dodonaea*, *Lannea*, *Phoenix, Acacia*, and *Cocos*), of which many were pronounced along the first two PC axes. Results of leave-one-out cross validation also indicated that only one sample was misclassified for each of the tested factors: Month (“CGn08”), Year (“GTn09”), and Location (“SJa09”). Two out of 3 misclassified samples were designated as multi-floral origin. The analysis helped delineate the pollen taxa of importance *vis-à-vis* the considered factors, found in both high and low proportions in the honey samples.

## Discussion

Our results showed that pollen spectra were equally comparable between Locations and also between Months and Years; the importance of this result, is that it helped to demonstrate the complexity of ecological/environmental phenomena involved in the process of foraging by bees in a heterogeneous and complex landscape. This shows that single, random samples of honey are not likely to provide reliable replicates of the pollen spectra. Furthermore, samples and taxa groups were well delineated based on the three factors considered; the importance of this result is that we now have a tool to classify additional pollen spectra, even when there is a low overall replicability.

The honey pollen content reflected the vegetation characterized by Tropical Dry Evergreen Forest (TDEF) species typical of Coromandel Coast [42]–[44]; markers were both predominant (*e.g.*, *Lannea*, *Dodonaea*, and *Mollugo*) and less-represented (*e.g.*, Melastomataceae/Combretaceae, *Drypetes* and *Glycosmis*). Taxa such as *Lannea*, *Dodonaea*, *Cocos* and *Borassus* have been reported to be foraged by the same bee species in other parts of south India [8], [45]. Some taxa such as Poaceae and Cyperaceae, generally reported in very low proportions, were frequently found in our samples, sometimes in considerable proportions. The present study, one of the first comprehensive melissopalynological contributions to the TDEF in the context of plant-pollinator interaction, is unique in that it documents, over time and space, differences in the pollen contents of honey, even within the confined landscape mosaic. At larger spatial sampling scales, this method is likely to throw up even more differences. As floral availability in the immediate vicinity of the beehive was ensured at all sites during most seasons, we assumed that the bees did not go into neighbouring sites to forage.

Bees are considered to be predominant pollen vectors in tropical forests [46]–[49], yet studies limited to bees in Southeast Asia are rare [48], thus there is an urgent need for forest bee community level data [50] for natural regeneration and restoration.

We found 80 pollen taxa from 42 samples. In a compilation of honey collected across a few hundred kilometers of Andhra Pradesh in south India, 104 taxa were reported from 164 samples [8]. In general, melissopalynological studies used random sampling because the main concern was determining the broad geographic origins of honey, which did not require long term monitoring. Thanks to our methodology of achieving high pollen counts, we report here, in a smaller geographic space, a comparable number of taxa, allowing us to exploit other statistical analyses.

We found a higher degree of similarity with qualitative index (bBC) and no structure with quantitative index (BC), probably due to the complex web of factors influencing the pollen content of honey. Apart from phenology and such other “external” ecological/environmental factors, factors related to bee behaviour such as the individual ability of some bees to remove more or less pollen from the nectar they collect [23] also come into play in this complexity. Similarity indices and ordination analyses were used to classify honey samples based on their spatial location over two provinces in Spain [20]. They found similar trends, the focus being botanical and again broad geographic origins. However, the temporal variations were not taken into account in that study. To our knowledge, in India only one study [51] has calculated qualitative indices on 6 samples collected in the same season; quantitative indices have not been reported for India.

Recent studies have effectively used similarity indices as well as ordination techniques such as PCA and LDA [21], [52]–[53] to classify the honey samples in terms of their botanical and geographic origins. Because of the temporal dimension in our study adding MANOVA was useful. Combining multivariate techniques (MANOVA, PCA, and LDA) with the pollen counts helped us to classify the samples in terms of factorial influences and understand the dynamics of bee foraging preferences.

Ordination analyses helped delineate pollen taxa such as *Dodonaea*, *Lannea*, *Phoenix* and *Acacia* collected by the bees in large proportions. Species corresponding to these taxa have a distinct flowering season in contrast with *Cocos*, which remained available through most seasons. Even during the peak flowering of these taxa, the bees continued to visit and gather pollen from *Cocos*. This finding supports the results of others [8], [45] regarding the bees' preference of *Cocos* pollen.

*Lannea* and *Dodonaea* were consistently recorded during summer and winter seasons, respectively, but their abundances varied inter-annually due to variations in rainfall. Seasonal variations in rainfall and soil water availability drive flowering periodicity [54]–[56]. In a tropical context, the physical condition of the sites, the neighboring vegetation, and the effect of animals influence the variation of individual phenology [57]. These may have reflected spatial differences in the pollen assemblages within the vegetation mosaic of our study area, which however seemed less pronounced than the other (temporal) factors like phenology. Thus, this method can be used for tracing floral phenology across years (scarce versus good flowering) and its effect on bees as the major pollinator in the community.

Discriminant analysis helped to highlight taxa found in small proportions in the honey, such as *Madhuca*, *Cassia*, *Grewia*, Commelinaceae and Malvaceae. Our direct observations [Ponnuchamy (2014), PhD Thesis, Pondicherry University, India] corroborate that they frequently visit the species corresponding to these taxa. Though frequently in low abundances, the consistent presence of these taxa in the honey suggests that, rather than serving as the bees' reward, they may actually be getting the benefit of pollination (by the bees). Some other studies [21]–[22], [53] also used discriminant analyses to highlight low abundance taxa. Though not in the purview of the present study, this highlights the need to quantify the pollen present in the nectar [58], for the tropical plant taxa, documented here as potential bee-plants (Table S1).

Though our analyses highlighted only a few outliers or misclassified samples, it seems likely that this may be a result of source scarcity or bees' preference. Some additional taxa with low discriminant power such as *Atalantia*, *Phoenix* and *Ziziphus* are among the broad geographic indicators of the Coromandel Coastal environment.

Part of the reason for the apparent wide variation in the pollen data results may be directly attributed to sampling a combination of both pollen cells and honey cells. We accept that such sampling could account for much of the differences found in the samples collected during different seasons as well as years. In other words, our results reflect not only the primary nectar sources of the bees but also the primary pollen collecting plant sources. Studies in other geographic areas [9], [46] have shown that the primary nectar and primary pollen foraging plants are usually different. It is true that sometimes in a hive a minor amount of pollen might be collected from a primary nectar source, as the bees eliminate much, but not all, of the pollen they collected with the nectar; the reverse is sometimes also true when the bee has no time to remove large quantities of pollen from the nectar, but rarely are the same plants used primarily for both aspects [7], [59]. However, our field observations in south India have shown that bees can source a) nectar alone from some plants (e.g., *Lawsonia inermis* L., *Neolamarckia cadamba* (Roxb.) Bosser and *Pongamia pinnata* (L.) Pierre) b) pollen alone from some plants (e.g., *Cocos nucifera* L., *Oryza sativa* L. and *Peltophorum pterocarpum* (DC.) K. Heyne) and c) pollen and nectar from some plants (e.g., *Haematoxylum campechianum* L., *Derris ovalifolia* (Wight & Arn.) Benth. and *Dodonaea viscosa* (L.) Jacq.,). The other aspect is that bees are opportunists that often gather pollen from plants that are easily accessed from their hives and as opportunists, choose plants that can provide them both nectar and pollen. This aspect could easily explain how the honey examined from our study area in South India contained ample amounts of anemophilous pollen from plants that are not used for nectar sources. Those pollen undoubtedly came from the pollen storage cells that were incorporated into honey samples that were studied.

A potential future experimental design is a small tweak to the methodology: to separately collect and process 1) only the pollen cells from the brood comb 2) only the capped honey cells from the brood comb and c) only capped honey cells from the super, with the hypothesis of a higher overall replicability.

Spatial and temporal factors as well as bees' preferences can play a vital role in honey pollen influx, which ultimately affects replicability. Results show that melissopalynological sampling in conjunction with multivariate statistics is a powerful tool to help classify honeys and understand the foraging preferences of bees in space and time. Our methodology also allowed us to assess replicability, which underlined the need for systematic studies incorporating both the spatial and temporal dimensions, especially in tropical conditions. With an appropriate experimental design taking into account all the complexities of plant-pollinator interactions at a community level, melissopalynology has the potential to provide a holistic ecological perspective in generating data from several landscapes or within a landscape in the context of climate change or effects on the pollinator community or ecosystem functions and services.

## Supporting Information

Table S1 List of pollen taxa recorded during the analyses of 42 honey samples collected over a 3 year period (2007–2009) near Puducherry South India. Note: Herbs (H); Shrubs (SH); Trees (T); Climbers (C); Sedges (SE); Grasses (G); Epiphytes (E); Non-Classified (NC).
(DOC)
