# Supplementary material for: Honey Pollen: Using Melissopalynology to Understand Foraging Preferences of Bees in Tropical South India
Source: PLoS One. 2014 Jul 8;9(7):e101618. doi: 10.1371/journal.pone.0101618 (PMC4086892; doi:10.1371/journal.pone.0101618)
Supplement: Table S1 — List of pollen taxa recorded during the analyses of 42 honey samples collected over a 3 year period (2007–2009) near Puducherry South India. Note: Herbs (H); Shrubs (SH); Trees (T); Climbers (C); Sedges (SE); Grasses (G); Epiphytes (E); Non-Classified (NC). (DOC) [file pone.0101618.s001.doc]

**Table S1** List of pollen taxa recorded during the analyses of 42 honey samples collected over a 3 year period (2007-2009) near Puducherry South India *Note: Herbs (H); Shrubs (SH); Trees (T); Climbers (C); Sedges (SE); Grasses (G); Epiphytes (E); Non-Classified (NC).*

| **No** | **Family** | **Pollen taxa** | **Life form** |
| --- | --- | --- | --- |
| 1 | Acanthaceae | *Blepharis* | H |
| 2 | Acanthaceae | *Justicia* | H |
| 3 | Acanthaceae | *Strobilanthes* | SH |
| 4 | Aizoaceae | *Mollugo* | H |
| 5 | Anacardiaceae | *Anacardium* | T |
| 6 | Anacardiaceae | *Buchanania* | T |
| 7 | Anacardiaceae | *Lannea* | T |
| 8 | Anacardiaceae | *Mangifera* | T |
| 9 | Arecaceae | *Borassus* | T |
| 10 | Arecaceae | *Cocos* | T |
| 11 | Arecaceae | *Phoenix* | T |
| 12 | Bombacacee | *Bombax* | T |
| 13 | Bignoniaceae | Bignoniaceae | T |
| 14 | Boraginaceae | *Coldenia* | H |
| 15 | Cactaceae | Cactaceae | SH |
| 16 | Caesalpiniaceae | *Caesalpinia* | SH |
| 17 | Caesalpiniaceae | *Cassia* | NC |
| 18 | Caesalpiniaceae | *Delonix*/*Peltophorum* | T |
| 19 | Caesalpiniaceae | *Tamarindus* | T |
| 20 | Casuarinaceae | *Casuarina* | T |
| 21 | Celastraceae | *Maytenus* | SH |
| 22 | Chenopodiaceae/ Amaranthaceae | Cheno/Amar | H |
| 23 | Commelinaceae | Commelinaceae | H |
| 24 | Compositae | Compositae echinate | H |
| 25 | Compositae | Compositae fenestrate | H |
| 26 | Compositae | *Xanthium* | H |
| 27 | Convolvulaceae | Convolvulaceae | H |
| 28 | Convolvulaceae | *Evolvulus* | H |
| 29 | Cyperaceae | Cyperaceae | SE |
| 30 | Euphorbiaceae | *Acalypha* | H |
| 31 | Euphorbiaceae | *Bridelia* | T |
| 32 | Euphorbiaceae | *Croton* | H |
| 33 | Euphorbiaceae | *Drypetes* | SH |
| 34 | Euphorbiaceae | *Glochidion* | T |
| 35 | Euphorbiaceae | *Mallotus* | T |
| 36 | Euphorbiaceae | *Phyllanthus* | H |
| 37 | Euphorbiaceae | *Ricinus* | SH |
| 38 | Euphorbiaceae | *Securinega* | SH |
| 39 | Fabaceae | Fabaceae | NC |
| 40 | Lamiaceae | Lamiaceae | H |
| 41 | Lecythidaceae | *Careya* | T |
| 42 | Liliaceae | Liliaceae | H |
| 43 | Loranthaceae | Loranthaceae | E |
| 44 | Lythraceae | *Lagerstroemia* | T |
| 45 | Malpighiaceae | *Hiptage* | H |
| 46 | Malvaceae | Malvaceae | H |
| 47 | Melastomataceae/ Combretaceae | MelastCombr | T |
| 48 | Meliaceae | Meliaceae | T |
| 49 | Menispermaceae | Menispermaceae | C |
| 50 | Mimosaceae | *Acacia* | T |
| 51 | Mimosaceae | *Mimosa pudica* | H |
| 52 | Mimosaceae | *Prosopis* | SH |
| 53 | Moraceae/ Urticaceae | Moraceae/ Urticaceae | T |
| 54 | Myrtaceae | *Eucalyptus* | T |
| 55 | Myrtaceae | *Eugenia* | T |
| 56 | Myrtaceae | *Syzygium* | T |
| 57 | Oleaceae | *Olea glandulifera* | T |
| 58 | Poaceae | *Oryza sativa* | G |
| 59 | Poaceae | Poaceae | G |
| 60 | Rhamnaceae | Rhamnaceae | SH |
| 61 | Rubiaceae | *Borreria* | H |
| 62 | Rubiaceae | *Canthium* | SH |
| 63 | Rubiaceae | *Haldina* | T |
| 64 | Rubiaceae | *Ixora* | T |
| 65 | Rubiaceae | *Morinda* | T |
| 66 | Rubiaceae | *Randia* | SH |
| 67 | Rubiaceae | Rubiaceae | NC |
| 68 | Rubiaceae | *Wendlandia* | T |
| 69 | Rutaceae | *Atalantia* | T |
| 70 | Rutaceae | *Clausena* | SH |
| 71 | Rutaceae | *Glycosmis* | SH |
| 72 | Rutaceae | Rutaceae | T |
| 73 | Rutaceae | *Toddalia* | C |
| 74 | Sapindaceae | *Cardiospermum* | C |
| 75 | Sapindaceae | *Dodonaea* | SH |
| 76 | Sapindaceae | *Schleichera* | T |
| 77 | Sapotaceae | *Madhuca* | T |
| 78 | Tiliaceae | *Grewia* | SH |
| 79 | Ulmaceae | *Holoptelea* | T |
| 80 | Verbenaceae | *Clerodendrum* | SH |
